# Lobular capillary hemangioma in a patient with ankylosing spondylitis using adalimumab: an exuberant presentation^☆^^☆☆^

**DOI:** 10.1016/j.abd.2019.02.004

**Published:** 2019-10-26

**Authors:** Thadeu Santos Silva, Carlos Leonardo Martins Guimarães, Isabela Pimenta Xavier, Vitória Regina Pedreira de Almeida Rego

**Affiliations:** a Dermatology Service, Escola Bahiana de Medicina e Saúde Pública, Salvador, BA, Brazil; b Teaching and Care Outpatient Clinic, Escola Bahiana de Medicina e Saúde Pública, Salvador, BA, Brazil; c Pathology Service, Hospital Universitário Professor Edgard Santos, Universidade Federal da Bahia, Salvador, BA, Brazil; d Dermatology Outpatient Clinic, Hospital Universitário Professor Edgard Santos, Universidade Federal da Bahia, Salvador, BA, Brazil; e Dermatology Service, Hospital Universitário Professor Edgar Santos, Universidade Federal da Bahia, Salvador, BA, Brazil

**Keywords:** Granuloma, pyogenic, Hemangioma, capillary, Spondylitis, ankylosing, Vascular neoplasms

## Abstract

Lobular capillary hemangioma or pyogenic granuloma is a benign vascular tumor of the skin or mucous membranes. Most patients present a single lesion. It manifests clinically as an erythematous, friable, and fast-growing tumor. This report details a case with exuberant presentation in a patient with ankylosing spondylitis, using adalimumab. Factors triggering pyogenic granuloma are not well known. They may spontaneously regress, but most require treatment.

## Introduction

Lobular capillary hemangioma, also known as pyogenic granuloma, is a common benign vascular tumor in the skin and mucous membranes. Clinically, lobular capillary hemangioma usually presents as solitary exophytic growth, which may be pedunculated or sessile, with a lobulated or smooth surface.^1^ It is most common in children, but it can also develop in adolescents and young adults. The gums, lips, mucosa of the nose, face, and fingers are the most frequently involved sites.^1^, ^2^, ^3^

## Case report

**Figure 1:**
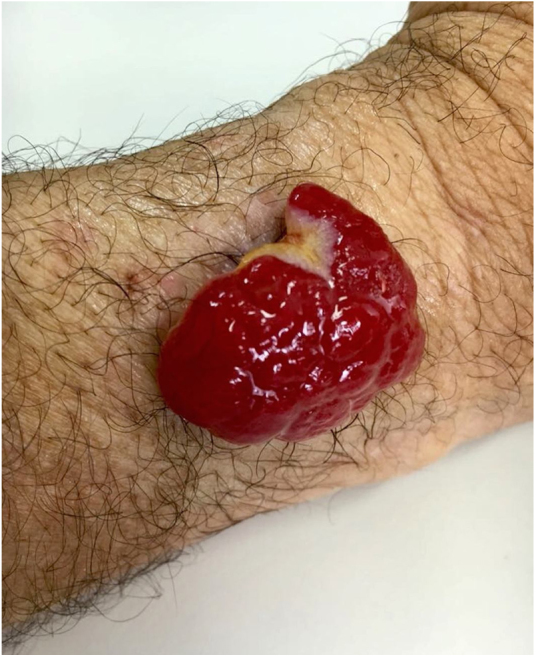
Exophytic erythematous nodule, measuring about 4.5 cm in left forearm.

**Figure 2:**
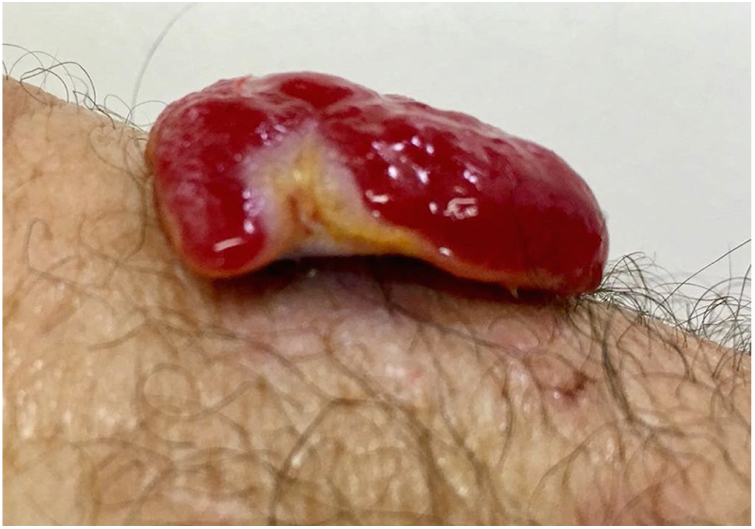
Pedunculated tumor.

**Figure 3:**
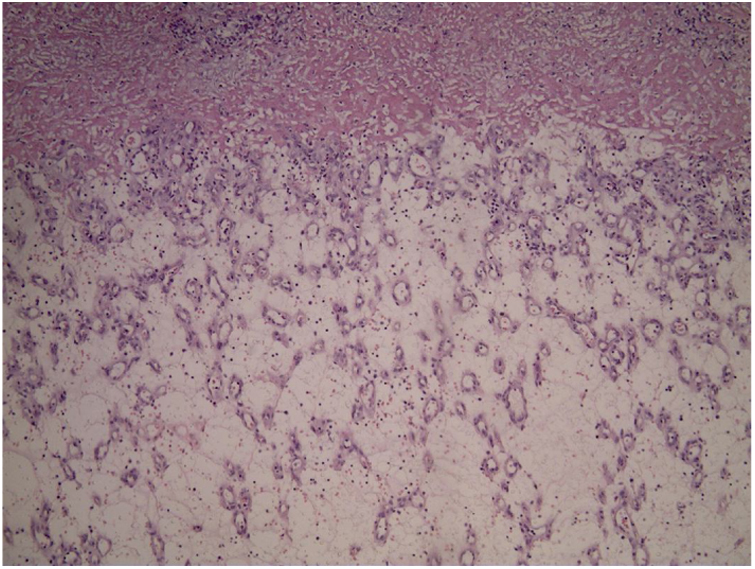
Overall appearance on histopathology: proliferation of small vessels (Hematoxylin & eosin, x40).

**Figure 4:**
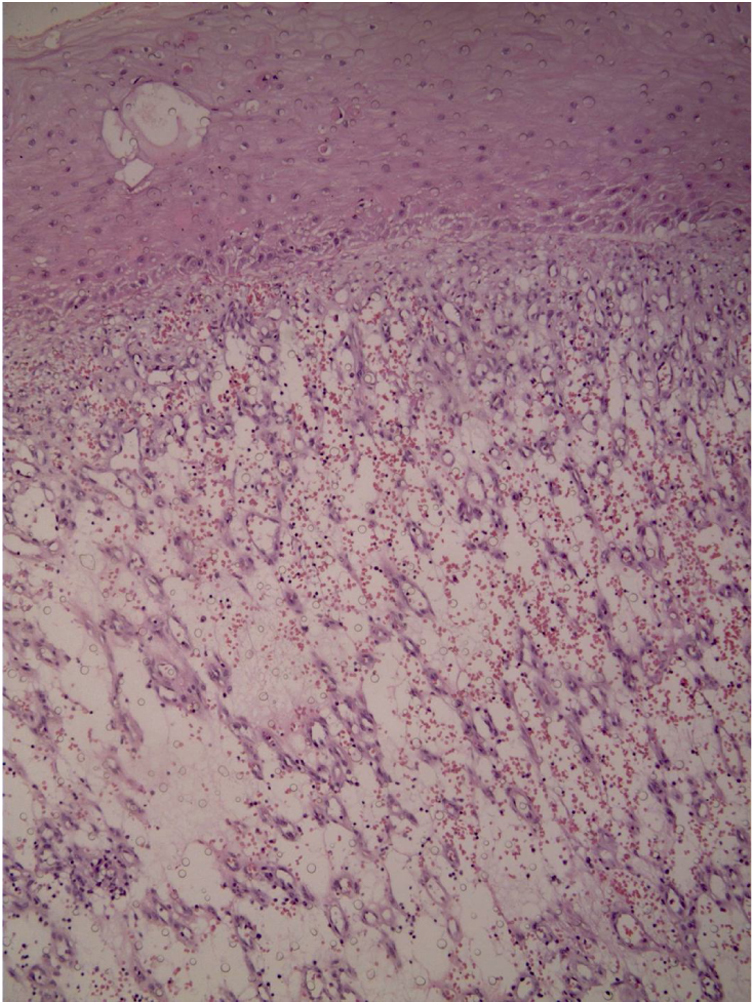
Histology: intense inflammatory infiltrate of neutrophils with fibrin deposition (Hematoxylin & eosin, x100).

A 45-year-old man with ankylosing spondylitis had been treated with adalimumab for five years. He reported an erythematous papule on the right forearm for four months, evolving with vegetative growth, fast-growing, friable, red-colored, measuring about 4.5 cm in diameter (Figure 1, Figure 2). He reported pain and episodes of spontaneous bleeding. The lesion was excised and the histopathological examination showed an ulcerated nodule. In the superficial dermis, there was a proliferation of small vessels and intense inflammatory infiltrate of neutrophils with fibrin deposition. In addition, foreign body giant cells phagocytosing refractory exogenous material were reported. In the deep dermis there was a proliferation of dilated capillaries in the midst of a plasmacytic and histiocytic inflammatory infiltrate, compatible with lobular capillary hemangioma (Figure 3, Figure 4). The patient maintains the use of adalimumab without the appearance of new lesions.

## Discussion

Factors triggering the lobular capillary hemangiomas are unknown and are often considered a hyperproliferative reactive vascular response to various stimuli, although they may also appear in healthy skin.^1^, ^4^ Factors considered predisposing include trauma, infection, inflammatory skin diseases, poor vascular formation, viral oncogenes, pregnancy, increased levels of female sex hormones, and neoplasms. The use of medications such as oral contraceptives, retinoids, epidermal growth factor inhibitors, and indinavir (a protease inhibitor) are associated with the appearance of lobular capillary hemangioma.^3^, ^4^, ^5^, ^6^ The use of tumor necrosis factor antagonists (anti-TNFs) has become a common practice in the treatment of various inflammatory diseases.^7^ There is a case report in the literature suggesting an association of pyogenic granuloma with the use of etanercept. The development of lobular capillary hemangioma might reflect the effects of angiogenic factors, such as vascular endothelial growth factor (VEGF), that are overexpressed in this lesion. TNF-α is able to induce keratinocyte expression, which, in turn, up-regulate VEGF production. The authors report a case of lobular capillary hemangioma due to an anti-TNF-α drug.^8^

## Conclusion

In most cases, treatment requires some therapeutic intervention. Local recurrence after incomplete excision or cryotherapy is common. Finally, ablative laser, shaving associated with electrocoagulation, or excision are procedures that show good effects.

## Financial support

None declared.

## Author's contributions

Thadeu Santos Silva: Statistical analysis; obtaining, analyzing and interpreting the data; effective participation in research orientation; critical review of the manuscript.

Carlos Leonardo Martins Guimarães: Approval of the final version of the manuscript; effective participation in research orientation; intellectual participation in propaedeutic and/or therapeutic conduct of the cases studied; critical review of the literature.

Isabela Pimenta Xavier: Statistical analysis; approval of the final version of the manuscript; conception and planning of the study; elaboration and writing of the manuscript; critical review of the manuscript.

Vitória Regina Pedreira de Almeida Rego: Statistical analysis; approval of the final version of the manuscript; elaboration and writing of the manuscript; obtaining, analyzing and interpreting the data; critical review of the literature; critical review of the manuscript.

## Conflicts of interest

None declared.
